# Small-angle X-ray scattering tensor tomography: model of the three-dimensional reciprocal-space map, reconstruction algorithm and angular sampling requirements

**DOI:** 10.1107/S205327331701614X

**Published:** 2018-01-01

**Authors:** Marianne Liebi, Marios Georgiadis, Joachim Kohlbrecher, Mirko Holler, Jörg Raabe, Ivan Usov, Andreas Menzel, Philipp Schneider, Oliver Bunk, Manuel Guizar-Sicairos

**Affiliations:** a Paul Scherrer Institut, 5232 Villigen PSI, Switzerland; b MAX IV Laboratory, Lund University, 221-00 Lund, Sweden; c Department of Physics, Chalmers University of Technology, 41296 Gothenburg, Sweden; d Institute for Biomedical Engineering, ETH and University Zurich, 8093 Zurich, Switzerland; e Bioengineering Science Research Group, Faculty of Engineering and the Environment, University of Southampton, Southampton SO17 1BJ, England

**Keywords:** small-angle X-ray scattering, tensor tomography, spherical harmonics, bone

## Abstract

Small-angle X-ray scattering tensor tomography, which allows reconstruction of the local three-dimensional reciprocal-space map within a three-dimensional sample as introduced by Liebi *et al.* [*Nature* (2015 ▸), **527**, 349–352], is described in more detail with regard to the mathematical framework and the optimization algorithm. For the case of trabecular bone samples from vertebrae it is shown that the model of the three-dimensional reciprocal-space map using spherical harmonics can adequately describe the measured data. The method enables the determination of nanostructure orientation and degree of orientation as demonstrated previously in a single momentum transfer *q* range. This article presents a reconstruction of the complete reciprocal-space map for the case of bone over extended ranges of *q*. In addition, it is shown that uniform angular sampling and advanced regularization strategies help to reduce the amount of data required.

## Abstract 2

The mathematical framework and reconstruction algorithm for small-angle scattering tensor tomography are introduced in detail, as well as strategies which help to reduce the amount of data and therewith the measurement time required. Experimental validation is provided for the application to trabecular bone.

## 1. Introduction   

Nanoscale features can be probed in a spatially resolved fashion when scanning a sample through a focused X-ray beam and recording a small-angle X-ray scattering (SAXS) pattern at each location (Fratzl *et al.*, 1997 ▸), a technique referred to as scanning SAXS. The real-space resolution is defined by the beam size and the scanning step size, which is typically chosen in the order of several to a few tens of micrometres, but can also be as small as some tens of nanometres or as large as millimetres. For each scanning point, nanoscale features are probed in reciprocal space by the SAXS pattern, typically measuring a spectrum of feature sizes in the range of a few nanometres to a few hundred nanometres. The capability to study the distribution of nanoscale structures over extended sample areas is of particular interest for hierarchically structured materials for which the length scales of interest span many orders of magnitude (Fratzl & Weinkamer, 2007 ▸; Meyers *et al.*, 2008 ▸; Beniash, 2011 ▸; He *et al.*, 2015 ▸; Van Opdenbosch *et al.*, 2016 ▸).

**Figure 1:**
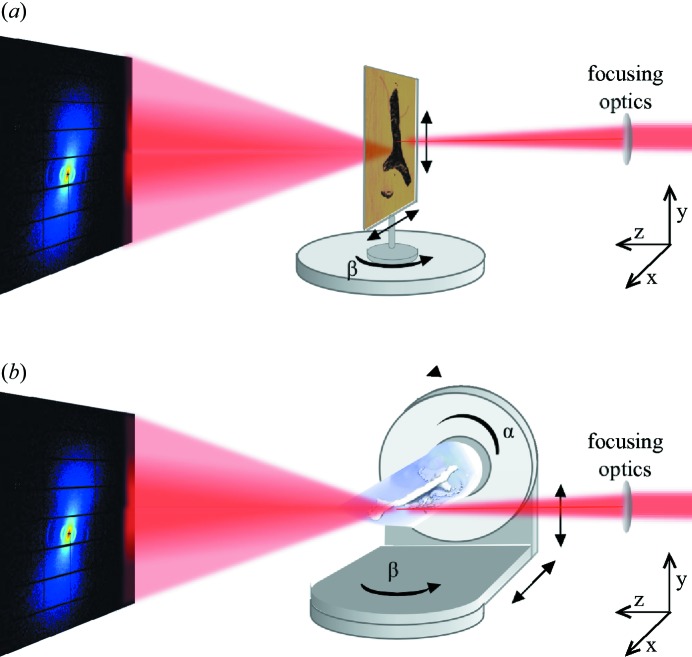
(*a*) Three-dimensional scanning SAXS setup to measure thinly sliced samples. The sample is scanned through the focused beam in *x* and *y* at different rotation angles β around *y*. (*b*) SAXS tensor tomography setup to measure three-dimensional samples. The sample is scanned through the focused beam in *x* and *y* at *n* orientations which are described by the rotation matrix 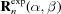.

A two-dimensional SAXS pattern contains statistical information about the nanostructure within the illuminated sample volume. If a main direction of orientation of the nanostructure exists, it can be determined in a model-independent way. Since a single SAXS pattern probes only a two-dimensional section through the three-dimensional reciprocal space, it provides incomplete information if the nanostructure is anisotropic in three dimensions. The three-dimensional orientation can be retrieved from thin sections of a sample by measuring SAXS patterns at different rotation angles, as illustrated in Fig. 1 ▸(*a*) (Liu *et al.*, 2010 ▸; Seidel *et al.*, 2012 ▸; Georgiadis *et al.*, 2015 ▸). To measure three-dimensional samples without sectioning, scanning SAXS has to be combined with computed tomography (CT) techniques. If the scattering is invariant with respect to sample rotation (Feldkamp *et al.*, 2009 ▸), measurements taken at different angles for one rotation axis are enough and a standard reconstruction algorithm, such as filtered back-projection or simultaneous algebraic reconstruction technique (SART), can be used. Rotational invariance applies for isotropically scattering samples, *i.e.* with no preferred orientation of the nanostructure or for unidirectional orientation of the nanostructure parallel to the rotation axis (Schroer *et al.*, 2006 ▸; Feldkamp *et al.*, 2009 ▸; Jensen *et al.*, 2011 ▸).

Skjønsfjell *et al.* (2016 ▸) have shown that, with strict assumptions on the sample, the orientation distribution can be retrieved from measurements using a single rotation axis by fitting a model of the X-ray scattering to the experimental SAXS pattern. For more general anisotropically oriented scatterers, SAXS acquisition using two rotation axes is needed (see Fig. 1 ▸ *b*) in order to retrieve the full three-dimensional reciprocal-space map (Liebi *et al.*, 2015 ▸; Schaff *et al.*, 2015 ▸). Schaff *et al.* (2015 ▸) have extended the concept of the rotation invariance (Feldkamp *et al.*, 2009 ▸) by the introduction of virtual tomographic axes. After sorting the two-dimensional SAXS patterns acquired at different rotations around two axes into over a thousand virtual tomography axes, standard reconstruction algorithms, here SART, are used to reconstruct the scattering parallel to each virtual tomographic axis independently.

Liebi *et al.* (2015 ▸) introduced a method based on the modelling of the three-dimensional reciprocal-space map of each voxel, *i.e.* volume element, using spherical harmonics and minimizing the error between the measured and modelled intensity by an optimization algorithm. The reconstruction contains in each voxel not only a scalar value, such as the X-ray attenuation coefficient in standard CT, but a tensor representing the reciprocal-space map; thus the method is referred to as SAXS tensor tomography. We present here in more detail the mathematical framework of SAXS tensor tomography, as introduced by Liebi *et al.* (2015 ▸), and the experimental validation of the model demonstrated on trabecular bone samples. We further investigate the angular sampling and the number of projections required for the reconstruction. In addition, some advanced regularization strategies are introduced and discussed, which reduce the amount of data and measurement time needed.

## 2. Model of the scattering signal using a three-dimensional reciprocal-space map   

**Figure 2:**
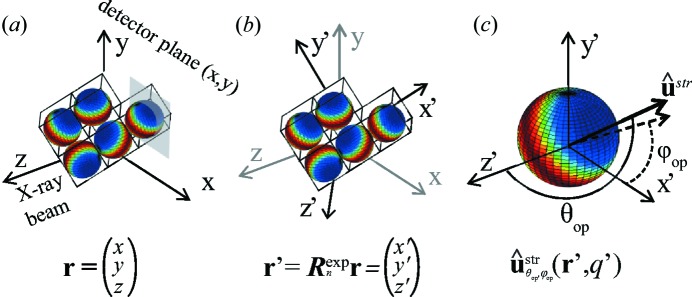
(*a*) Laboratory coordinate system 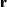 and (*b*) object-coordinate system 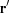, which are related by the rotation matrix 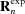. In (*a*) the planar two-dimensional cut through the reciprocal-space map in the plane of the detector is illustrated in grey. The object-coordinate system 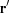 defines the position of each voxel of the object. (*c*) In each voxel a series of spherical harmonics functions is used to describe the three-dimensional reciprocal-space map, from which the scattering signal can be obtained. The preferential orientation of the nanostructure in each voxel and each *q* is characterized by a unit vector 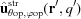 defined by the polar 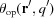 and azimuthal 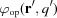 angles.

The *ab initio* characterization of the anisotropy of nano­strucure in a three-dimensional volume requires measurement of the sample from many possible orientations, not only around one rotation axis, but in a grid of two-dimensional orientation angles using two rotation axes. It is necessary to define a coordinate transformation from laboratory coordinates 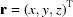, with *z* pointing along the direction of the X-ray beam, to sample Cartesian coordinates 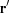, as illustrated in Fig. 2 ▸. This transformation is described for the *n*th sample orientation by a rotation matrix 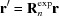.

The reconstruction aims to determine for each object-coordinate voxel, 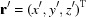, a three-dimensional reciprocal-space map, 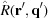, as a function of a reciprocal-space vector 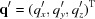. The object-coordinate system can also be described in spherical coordinates 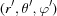, with 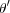 being the polar angle and 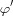 the azimuthal angle. In order to reach this objective, the reciprocal-space map is modelled using spherical harmonics 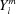 of degree *l* and order *m* (Jackson, 1999 ▸) as 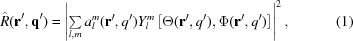
where 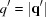 is the magnitude of the reciprocal-space vector, 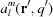 are the spherical harmonic coefficients, and 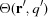 and 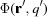 are the polar and azimuthal angles, respectively, which are arguments of the spherical harmonic function. The square of the sum is used to avoid non-physical negative intensities. Since spherical harmonics form a complete basis, higher orders can be used to model the scattering of arbitrarily complex nanostructures. For example, spherical harmonics were used in texture analysis of pole figures from X-ray diffraction measurements decades ago (Roe & Krigbaum, 1964 ▸; Bunge & Roberts, 1969 ▸) using orders up to 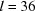 (Van Houtte, 1983 ▸). When reconstructing a scattering model for a large number of voxels of an extended sample however, reducing the number of coefficients and thus minimizing the total number of optimization parameters is of great benefit. For cases where there is a preferential direction of ordering for the nanostructure it is useful to orient the zenith of the spherical harmonics to this direction independently for each voxel in each *q* range. This results in a sparser representation and reduces the number of coefficients needed to model the reciprocal-space map. This is a particularly good strategy in cases where the three-dimensional reciprocal-space map is not too far from spherical or ellipsoidal symmetry; thus only a few components are required in the decomposition of 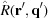 in equation (1). The local preferential orientation with respect to the object coordinates is characterized by a unit vector, 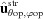 (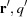), as illustrated in Fig. 2 ▸(*c*), which is parameterized by the angles 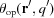 and 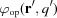. The arguments of the spherical harmonics, 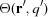 and 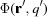, can be implicitly defined by 
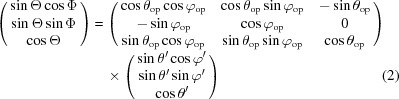
where 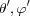 are the spherical coordinates of the object-coordinate system. Neglecting multiple-scattering effects, the contribution of each voxel to the intensity measured at the detector corresponds to a section through the three-dimensional reciprocal space along the Ewald sphere of radius 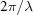. For the case of SAXS an expansion of zero order of the Ewald sphere leads to a planar two-dimensional cut through the reciprocal-space map, *i.e.* 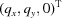, in the laboratory coordinate Cartesian system as illustrated in Fig. 2 ▸(*a*). The data in the plane 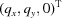 correspond to the 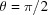 plane or 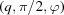 in spherical coordinates. In order to calculate the total intensity at the detector for the *n*th sample orientation, the contribution from all voxels along the beam path *z* is summed up, 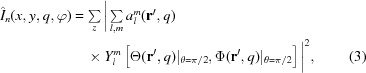
where the measured intensity for the *n*th sample orientation, 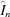, is a function of the scanning position 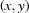.

## 3. Optimization algorithm   

For the reconstruction of the three-dimensional reciprocal-space map in one specific *q* range, an iterative optimization algorithm is used to minimize the error metric 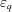 between the modelled intensity, 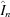 as calculated in equation (3), with respect to the measured data 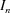, for all scanning positions 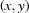 and sample orientations, 
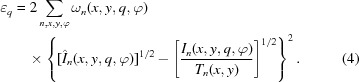
Minimizing this error metric is a first-order approximation to a maximum-likelihood estimation for photon-counting Poisson noise (Thibault & Guizar-Sicairos, 2012 ▸). The measured intensity at each point and each orientation is divided by the transmitted intensity 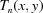 to compensate for absorption of the sample (Schroer *et al.*, 2006 ▸). The parameters to optimize are the spherical harmonic coefficients, 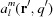, and their orientation through 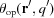 and 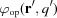. A binary mask, 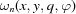, is used to denote valid data regions. It is equal to one for all valid data points and can be set to zero for bad detector angular sectors, φ, or where the scattering of the sample is obstructed, for example by the sample mount.

To compute 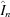 we first evaluate the sum of the different spherical harmonic terms and compute the modulus squared. The resulting quantity is then projected along the direction of the X-ray beam. The projection operation is calculated using bilinear interpolation. The resulting image is convolved with a 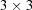 pyramidal kernel, which emulates the effect of up- and subsequent down-sampling, to reduce artifacts associated with the discrete volume grid. For the minimization of the error metric, given in equation (4), a conjugate gradient algorithm was used (Press *et al.*, 2007 ▸). As the number of optimization parameters is very large, the gradient of the error metric with respect to the optimization parameters is calculated analytically (see Appendix *A* [App appa]). In each iteration step the gradient is computed according to equations (10), (11) and (14), followed by a line search in a conjugate direction induced by the local curvature of the function to be optimized [equation (4)].

**Figure 3:**
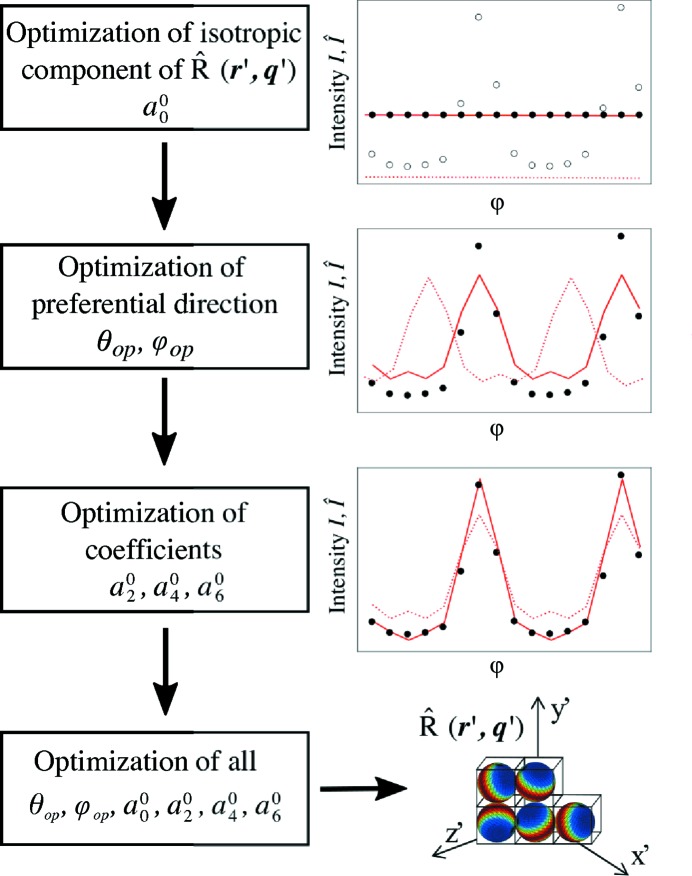
Flowchart of the optimization procedure. The output of the optimization is for each object-coordinate voxel 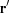 the three-dimensional reciprocal-space map 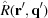 parameterized by the preferential orientation 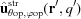 and the coefficients of the spherical harmonics 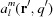. The optimization is performed in four steps to accelerate convergence. Red dotted lines indicate starting values of the modelled intensity 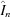 in each step, whereas solid red lines indicate the resulting 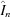 after the corresponding optimization step. The measured intensity 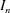 is shown as black dots; in the first step the optimization is performed using the data averaged over φ.

In order to accelerate convergence, the optimization is performed in four steps as illustrated in Fig. 3 ▸. First, the isotropic component of the reciprocal-space map 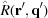 is optimized by using only the isotropic spherical harmonic term with 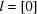 and 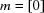 and averaging the data over φ. Secondly, only the angles are optimized and the coefficients for 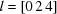, 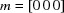 are kept constant at a factor *s* times the 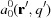 obtained in the first step. For the bone measurements shown in this paper the coefficients were set to 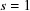 for 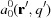, 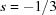 for 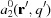 and 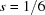 for 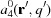. These coefficients were chosen to approximate the anisotropy which was observed in similar samples. Alternatively, symmetries in the three-dimensional reciprocal-space map, which help in determining the number of spherical harmonics functions needed, can be estimated from the shape of the scattering object studied. Without previous knowledge of the sample, by repeating the optimization procedure with different constant values of the coefficients in the second step and comparing the results, one can avoid a solution being obtained which is just a local minimum of the error metric. It is also possible to gain knowledge about the three-dimensional reciprocal-space map by studying a flat sample at different sample rotations as outlined in §4. In the third step the co­efficients 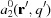, 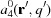 and 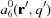 were optimized while the angles and 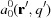 from the first step were kept constant. Finally, in the last step all coefficients and angles were optimized simultaneously.

A support constraint on the object was used in order to optimize only the part of the object three-dimensional grid volume which contains the sample. For this a three-dimensional binary support mask, 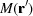, is introduced, denoting with zeros the regions without sample. This can be obtained, for example, using a threshold after the first optimization step where a first estimate of 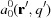 is obtained. The mask, 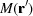, is then used when computing the gradients to set the gradients outside the object to zero, as shown in equations (10), (11) and (14). Boundary conditions on the values of the coefficients can be introduced either using constrained optimization algorithms or by introducing a smooth penalty term to the error metric. This can be used for example to enforce symmetries if it is known that they are present in the sample. For example, for the case of 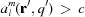


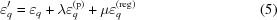
where 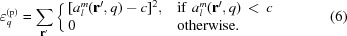
Here λ controls the strength of the penalty term. In the reconstructions shown in the article, no such penalty term was used. The regularization term, 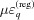, in equation (5) will be explained in §5.2.

## 4. Experimental validation of the reciprocal-space model for trabecular bone   

To validate the suitability of a series of spherical harmonics as a model to describe the three-dimensional reciprocal-space map, we used data from a 20 µm-thin section of trabecular bone (sample A). The data were taken with a beam size of 20 × 20 µm at different rotation angles β around the *y* axis of the beamline coordinate system as illustrated in Fig. 1 ▸(*a*). Further experimental details can be found in Appendix *B* [App appb]. As the lateral resolution matches the thickness, the measurements give an adequate representation of imaging a planar arrangement of voxels and it has been shown that for thin samples a single rotation axis provides sufficient information on the three-dimensional arrangement of nanostructures (Georgiadis *et al.*, 2015 ▸). Image registration (Guizar-Sicairos *et al.*, 2008 ▸) of the transmission images, recorded simultaneously to the SAXS data, was used to assign the scattering from multiple orientations to individual voxels. This procedure is described in more detail by Georgiadis *et al.* (2015 ▸).

**Figure 4:**
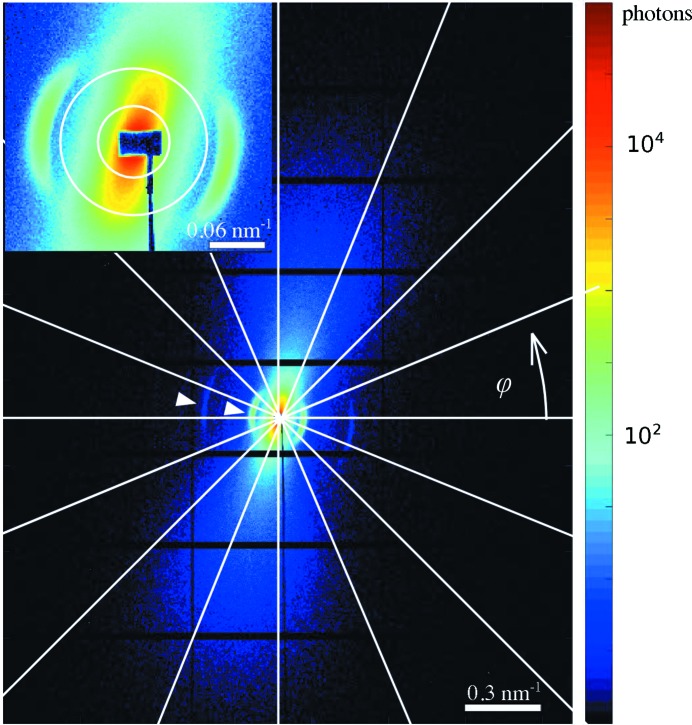
Two-dimensional scattering pattern in logarithmic scale obtained from a thin slice of trabecular bone and a zoom-in to the low-*q* range. The 16 segments in which the radial integration is performed are indicated by white lines and two circles that mark a range of *q* values. White triangles point at the pronounced first and the faint third diffraction orders of mineralized collagen fibres.

Fig. 4 ▸ shows a single scattering pattern from one scan point of the measurement. Since the nanostructure causing the scattering has a preferential orientation, the recorded SAXS pattern is anisotropic. Furthermore, since the nanostructure anisotropy is three dimensional, the scattering pattern depends on the sample orientation, β (Georgiadis *et al.*, 2015 ▸). The scattering from bone originates mainly from the electron-density contrast between hydroxyapatite mineral crystal platelets and the surrounding material with lower electron density, such as collagen and water (Fratzl *et al.*, 1997 ▸). The main characteristics of the two-dimensional SAXS signal from bone are the distinct Bragg reflections from the periodic gaps in the collagen fibrils which are filled by hydroxyapatite crystals (Fratzl *et al.*, 1991 ▸). The repeat distance of these gaps is approximately 65 nm and they produce characteristic arcs in the scattering pattern (Wilkinson & Hukins, 1999 ▸). The first and third harmonic of this signal are indicated by white triangles in Fig. 4 ▸. In the direction perpendicular to these arcs a fan-shaped scattering profile can be seen, which arises from the shape, size and lateral arrangement of the mineral platelets in and around the collagen fibrils (Norio *et al.*, 1982 ▸). At high *q* values, *q* > 0.125 nm^−1^, primarily the mineral platelets with an approximate size of 3 × 25 × 50 nm can be probed. At low *q* values, *q* < 0.1 nm^−1^, the scattering contains mainly information on the lateral arrangement of the collagen fibrils and the diameter of the fibrils, which is typically in the range of 50–200 nm (Fratzl & Weinkamer, 2007 ▸; Gourrier *et al.*, 2010 ▸; Pabisch *et al.*, 2013 ▸; Giannini *et al.*, 2014 ▸). The organization of bone is hierarchical: the orientation of the mineral crystals, the collagen fibrils, the fibril bundles, the fibres and the macroscopic bone organization are closely related to each other (Rinnerthaler *et al.*, 1999 ▸; Fratzl & Weinkamer, 2007 ▸; Seidel *et al.*, 2012 ▸; Granke *et al.*, 2013 ▸; Georgiadis *et al.*, 2015 ▸, 2016 ▸).

**Figure 5:**
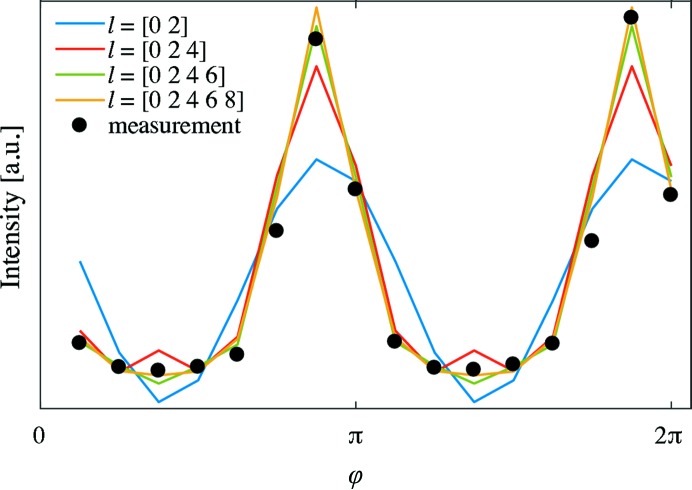
SAXS measurements from a single voxel for *q* = (0.0379–0.0758 nm^−1^) for one sample rotation angle β = 20° are shown with black dots. The corresponding modelled data (lines) with different degrees *l* and zero order *m* of spherical harmonics are shown as a function of the azimuthal angle φ in the detector plane.

The intensity of each scattering pattern was integrated in 16 azimuthal segments, shown by white lines in Fig. 4 ▸, for each momentum transfer *q*. Increasing the number of azimuthal segments was found to have no significant influence on the results while increasing the amount of data and calculation time. Black dots in Fig. 5 ▸ show the measured intensity at the detector *versus* azimuthal angle φ in one *q* range, 0.0379–0.0758 nm^−1^, indicated by concentric circles in Fig. 4 ▸ in the 16 azimuthal segments. Symmetries, such as point symmetry, are very common in SAXS and can be easily enforced in our model by choosing the appropriate degrees and orders of the spherical harmonics. For the case of trabecular bone we assume a point symmetry around 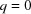 and a rotational symmetry around one axis. Thus, using only even degree *l* and zero order *m* is enough to model the scattering signal retrieved from the three-dimensional reciprocal-space map. If the mineral platelets did not point in all directions perpendicular to the fibril axis with equal probability, the resulting reciprocal-space map would not be a ring with cylindrical rotational symmetry. For this case in which the rotational symmetry is not given, higher azimuthal orders, *m*, of spherical harmonics are needed to capture this feature. Fig. 5 ▸ shows for one sample orientation, β = 20°, the intensity at the detector *versus* azimuthal angle φ, for the measured and modelled data. The azimuthal orientation of the scattering pattern can already be described well using only degrees 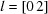. However the only functional dependence allowed for these small orders is a sine or cosine function (blue line in Fig. 5 ▸), which is insufficient to quantitatively model the scattering profile, as can be seen in Fig. 5 ▸. Using 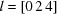 and 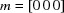 (red line) the sharpness of the peak is not yet described fully, and an artificial increase in the low-intensity region appears. Using spherical harmonics of degree 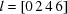 and order 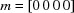 (green line) it is possible to reproduce reliably the measured data. Adding yet a higher degree (yellow line) does not improve the model significantly.

**Figure 6:**
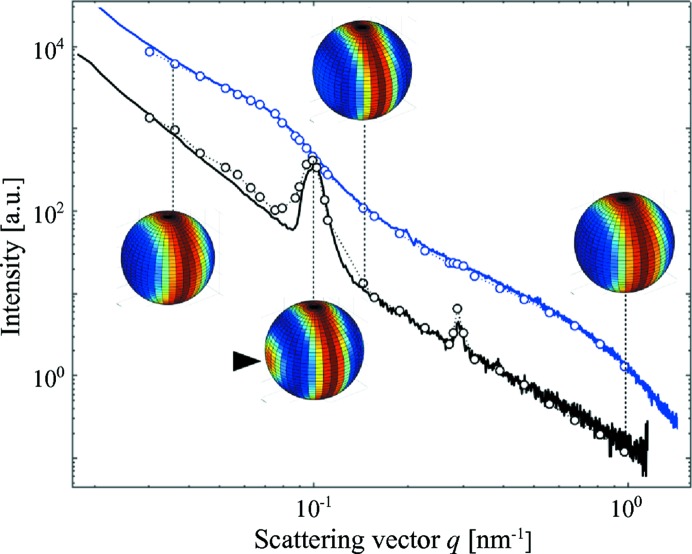
Plots for measured (solid lines) and modelled (circles and dashed lines) intensity from a single voxel in two of the 16 azimuthal segments on the detector plane. The optimization was performed in 32 *q* values separately. For some *q* values the three-dimensional reciprocal-space maps are shown mapped onto a sphere. A black triangle points towards the intensity cap characteristic of the 65 nm collagen repeat distance.

Consequently, the model was applied with 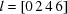 and 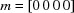 to reconstruct the *q*-resolved three-dimensional reciprocal-space map from the data set from sample A. The optimization was performed at 32 *q* values between 0.0303 and 0.984 nm^−1^, each with a radial width of 0.0061 nm^−1^. Fig. 6 ▸ shows a good agreement of the fit (points and dashed lines) with the data (solid lines) for the full *q* range covered by the two-dimensional detector. For selected *q* values the modelled three-dimensional reciprocal-space map is shown. The characteristic reciprocal-space footprint of the mineral platelets, which appears as a fan-shaped profile in a two-dimensional pattern as seen in Fig. 4 ▸, appears in the three-dimensional reciprocal-space map as a ring perpendicular to the direction of the fibril axis and with angular spread related to the fibrils’ degree of orientation. This ring can be observed in the whole investigated *q* range in Fig. 6 ▸. The Bragg reflection, which is associated with the collagen periodic gaps, appears in the three-dimensional reciprocal-space map as a cap. It is visible for the first and third harmonic of the reflection (see triangle in Fig. 6 ▸). Using 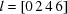 and 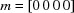 is enough to capture both features typical for the scattering from bone. Reconstructing the full *q*-resolved three-dimensional reciprocal-space map opens up the possibility of retrieving the size and shape of the scattering object from fitting, as done in standard small-angle scattering analysis (Bressler *et al.*, 2015 ▸).

## 5. SAXS tensor tomography   

In order to extend this method to volumetric samples we combine SAXS with CT. In standard CT a scalar quantity, such as the sample absorption, is measured for each point within two-dimensional projections and the reconstruction is three dimensional. In such a case it is sufficient to measure projections at different sample orientations around a single rotation axis which is perpendicular to the X-ray beam propagation direction. For the case of SAXS one needs a reconstruction of the three-dimensional reciprocal-space map for each voxel, as described by the six-dimensional function in equation (3). Using the principles of invariant scattering along the direction of sample rotation (Feldkamp *et al.*, 2009 ▸), Schaff *et al.* (2015 ▸) showed that for an *ab initio* reconstruction it is sufficient to measure SAXS patterns from all points of the sample, while sampling object orientations in the full 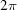 steradians. To achieve this in the experiment a second rotation axis was introduced as schematically shown in Fig. 1 ▸(*b*). Raster scans of the whole sample, here also referred to as projections, are measured at different angles α and different tilt angles of this rotation axis, β. The object rotation matrix 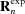 in each sample orientation *n* can be calculated by a rotation around *y* by an angle 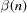 followed by a rotation around *x* by an angle 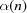, resulting in 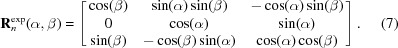
Because the sample is not perfectly aligned in the rotation centre of both rotation axes, the translational alignment of the measured projections has to be refined. For this purpose an X-ray absorption tomogram is reconstructed from the measured transmission images at β = 0°, using standard filtered back-projection algorithms. The sample transmission is measured simultaneously to the SAXS pattern using a photo-diode mounted on the beamstop, which blocks the direct unscattered beam and avoids damage to the detector. For each object orientation, (α, β), a projection from this absorption tomogram was calculated and used as a reference for alignment of the measured transmission images at the corresponding orientation. For this an efficient image registration approach based on selective up-sampling of the cross-correlation (Guizar-Sicairos *et al.*, 2008 ▸) was used. If the sample has little contrast in the absorption measurement, alternatively the scattering intensity, averaged over φ in a chosen *q* range, can be used for this step.

A human trabecular bone cylinder (sample B, Appendix *B* [App appa]) of about 1 mm^3^ was measured with a scanning step size in *x* and *y* of 25 µm. In total 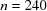 projections, *i.e.* different sample orientations 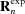, were measured with an angular step of 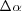 = 4.5° between 0 and 180° and 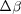 = 15° between −30 and 45°. The analysis was carried out following the procedure as described in §3 using a *q* range between 0.0379 and 0.0758 nm^−1^.

**Figure 7:**
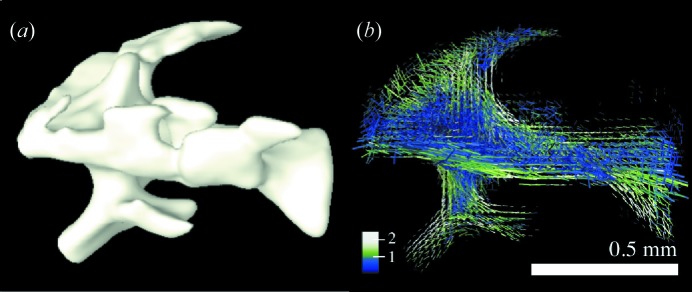
Human trabecular bone (sample B). (*a*) Standard tomographic reconstruction based on the sample X-ray absorption. (*b*) Orientation of the bone ultrastructure as retrieved from SAXS tensor tomography. Colour, length and direction represent degree of orientation, isotropic component 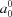 and main orientation of the collagen fibrils, respectively.

In Fig. 7 ▸ the resulting main orientation of the bone ultrastructure is indicated by the direction of cylinders, the strength of the isotropic component 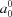 by the length of cylinders, and the degree of orientation ρ by the colour. The latter is calculated by the ratio of the anisotropic component of 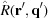 to 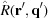, namely 
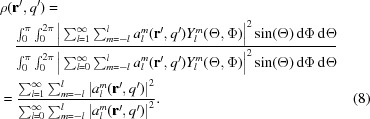
For the reconstruction shown in Fig. 7 ▸, 830 000 SAXS patterns were used, which were acquired in 8 h total exposure time and an overall measurement time of 20.3 h including overhead from motor movements. More details about the measurement are given in Appendix *B* [App appb]. Minimizing measurement time can be tackled by changes in the hardware, *e.g.* reducing motor scanning overhead and faster detectors (Tinti *et al.*, 2015 ▸). Another important aspect is to optimize spatial and angular sampling, as discussed in the following section.

### 5.1. Dependence of reconstruction quality on angular sampling   

**Figure 8:**
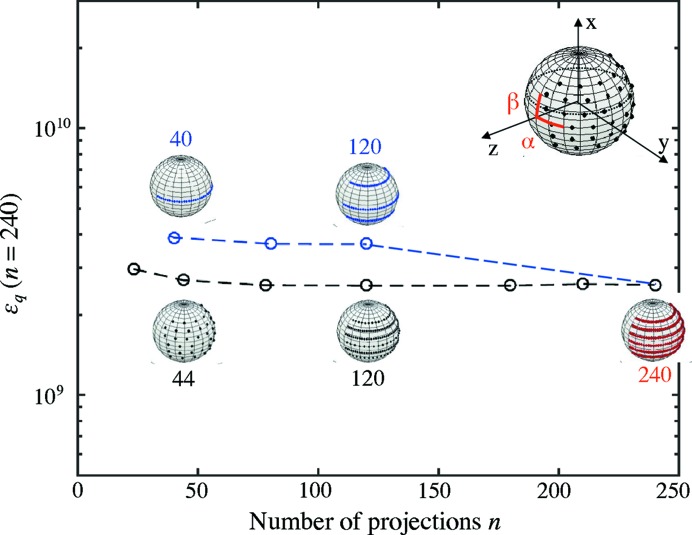
The error according to equation (4) between the modelled intensity and all measured projections (= 240) as a function of the number of projections used in the optimization. The two curves show the error metric for data sets where the projections are reduced by keeping constant either 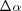 (blue) or 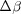 (black). The spherical insets show the angular sampling schematically drawn on a sphere, where each object orientation (α, β) can be represented by a point on the hemisphere.

In order to study the effect of the angular sampling of object orientations on the reconstruction, the optimization procedure for sample B was repeated using different subsets of the measured angular projections *n*. Whereas a smaller number of projections are used during these reconstructions, to compare the final quality of the reconstruction Fig. 8 ▸ shows the error with respect to all 240 projections. Therefore, the full data set is used as a ‘gold standard’ even when only a subset of the data is available in these test reconstructions. The whole data set is schematically shown in Fig. 8 ▸ (in red), where each point on the sphere represents a sample orientation and the measurements at the six values for β are shown as six meridional rows. For comparison, the projections from the full data set have been removed, keeping either the original 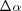 = 4.5° (in blue), or 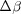 = 15° (in black).

For the blue curve, we obtain 120 projections by taking a subset of the data corresponding to 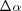 = 4.5° and 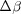 = 30°. Furthermore, 40 projections were obtained by removing completely the tilt of the rotation angles and keeping 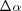 = 4.5°. In contrast, for the curve in black we label the result with 120 projections for which we increased 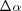 to 9° and kept the original 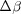 = 15°, and furthermore tested 44 projections by increasing 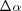 to 22.5° while keeping the original 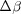 = 15°. That means the 40 projections (in blue) correspond to the sampling along only one rotation axis, similar to standard absorption-based CT, while the 44 projections (in black) correspond to an almost isotropic angular sampling (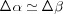).

The black curve with a more isotropic angular sampling results in a lower error compared with the corresponding points on the blue curve. The error using 120 projections around three tilt angles 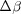 of the rotation axis in blue is even higher than for the 44-projection reconstruction with isotropic angular sampling (black), even though the latter has close to 60% fewer projections. This difference can be attributed to the angular distribution of these projections, where the 44-projection tomogram has almost isotropic angular sampling in α and β, and emphasizes the importance of adequate angular sampling of the full hemisphere of sample orientations.

**Figure 9:**
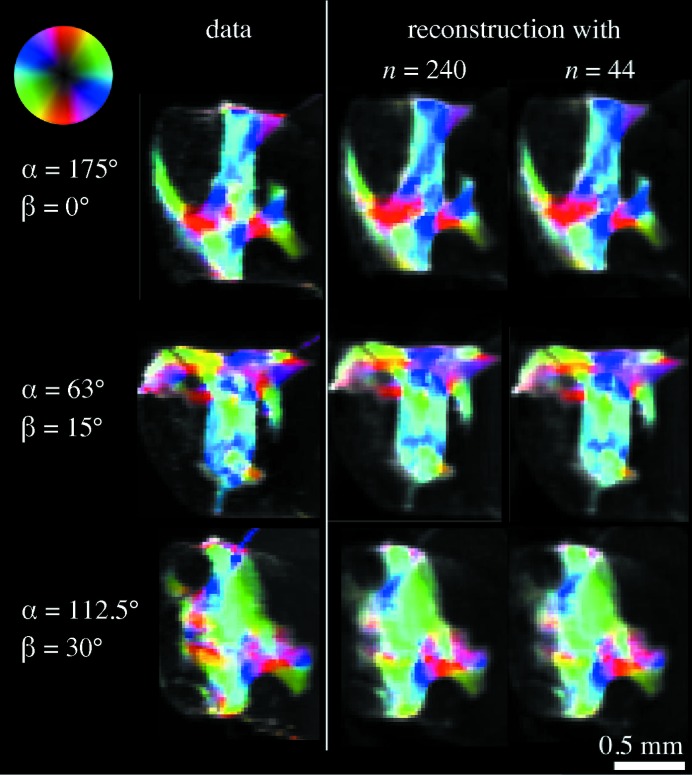
Comparison between measured two-dimensional scanning SAXS projections and the equivalent projection obtained from the reconstruction using subsets of the data corresponding to 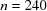 and 44 sample orientations. The colour wheel represents the main scattering orientation, the hue the scattering intensity, and the degree of orientation the colour saturation.

Following the black line, it is evident that the error metric with respect to the full data set remains constant for an extended range of sample rotations *n*, which means that the fit to the complete data set does not change much by reducing the number of projections, even to a fraction close to one third. This indicates that the data set was taken with significantly more projections than needed. One reason is that the choice of 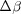 = 4.5° was based on the full diameter of the sample, not taking into account any sparsity of the sample, which could reduce angular sampling requirements. The spatial sparsity of the sample is actively used in the tensor tomography reconstruction through the mask 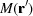. Another form of sparsity may arise from the fact that the bone ultrastructure exhibits domains in the order of a few hundred micrometres. In these reconstructions we did not use corresponding constraints. However this domain structure is evident even from the SAXS projections in Fig. 9 ▸. In §5.2 we show an approach to take advantage of this knowledge through regularization.

As a validation of the reconstruction, the two-dimensional projections from SAXS tensor reconstruction were computed and compared with the data; in other words we compared the measured scanning SAXS data for a projection in a given orientation of the sample, 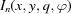, with the corresponding projected intensity of the reconstruction, 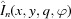. The first column in Fig. 9 ▸ shows a representation of a scanning SAXS projection where the scattering intensity, degree of orientation and main scattering orientation are mapped to the image intensity, colour saturation and hue, respectively (Bunk *et al.*, 2009 ▸). The colour-wheel inset relates the direction of the main scattering orientation to the particular hue. As already mentioned, it is evident that the trabecular bone sample exhibits domains within which the nanostructure orientation is spatially correlated. The projected intensity of the reconstructions is computed using equation (3) and shown in Fig. 9 ▸ for the case of the full data set (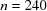) and for 44 projections. By examining Fig. 9 ▸ it can be seen that the experimental projections could be reliably reproduced with as few as 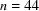 projections for this sample, which would effectively have allowed for a reduction to one fifth of the scanning time and the deposited dose.

### 5.2. Regularization strategies   

In some cases there is a tendency during a reconstruction to exacerbate high-spatial-frequency noise both in the coefficients 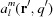 as well as in the direction 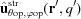. To alleviate this problem a regularization method was implemented. Regularization is often used in optimization to better constrain ill posed or ill conditioned problems. The chosen approach for the coefficients follows a method akin to Grenander’s method of sieves (Grenander, 1981 ▸) by spatially convolving the gradient with respect to the spherical harmonics coefficients [equation (10)] with a three-dimensional Hamming window. We use windows of either 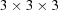 or 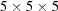 voxels. With this method the optimization solves first for the low and middle spatial frequencies while the high spatial frequencies are introduced last.

For the regularization of the local preferential orientation, 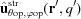, the gradient convolution approach is not suitable because the direction is jointly represented by two parameters, 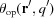 and 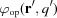. In addition, the representation of an orientation using a vector has the inherent ambiguity that 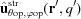 and 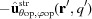 would represent the same local orientation for a reciprocal-space map with a point symmetry. These characteristics do not pair well with a convolution. To penalize spurious variations of the orientation from neighbouring voxels a regularization term, 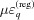, to the error metric was introduced in equation (5). The regularization term is based on the absolute value of the dot product between neighbouring 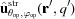, namely 
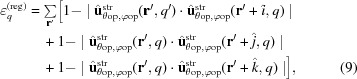
where 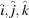 are the Cartesian coordinate unit vectors, pointing in 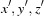, respectively. μ is a free parameter that controls the relative strength of the regularization.

**Figure 10:**
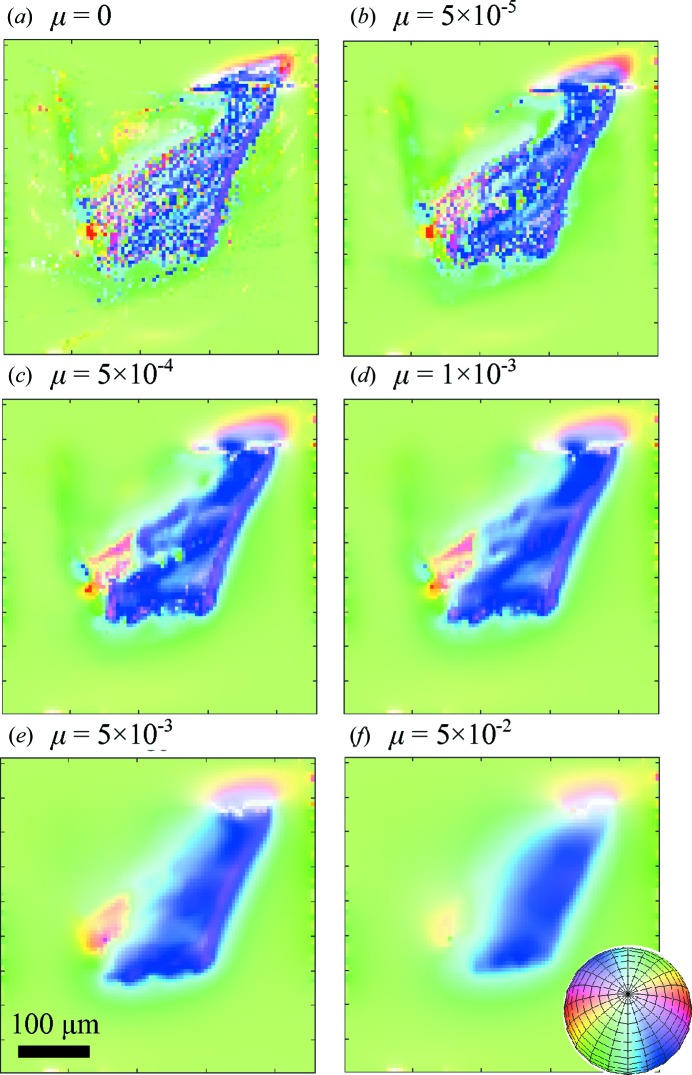
A cross section through the three-dimensional reconstruction of the local orientation, 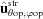, is shown for (*a*) a reconstruction without regularization and (*b*)–(*f*) with different values of the regularization parameter μ. The three-dimensional orientation of 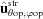 was encoded through both hue and saturation, as indicated by the inset colour sphere.

The effect of this regularization on the orientation is shown in Fig. 10 ▸. A trabecular bone sample (sample C) of approximately 250 µm diameter was measured with a beam of 5 × 1.4 µm and a scan step size of 5 µm with a total of 333 angular projections, with an even distribution of projection angles 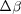 = 7.5° and 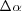 = 7.5° 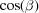 according to the discussion in §5.1. Fig. 10 ▸(*a*) shows an axial slice through the reconstruction of 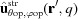 without regularization of the direction in which high-spatial-frequency fluctuations of the direction between neighbouring voxels are observed. Increasing the value of μ, Figs. 10 ▸(*b*)–10 ▸(*f*) demonstrate an increasing smoothness of the reconstruction of 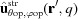.

**Figure 11:**
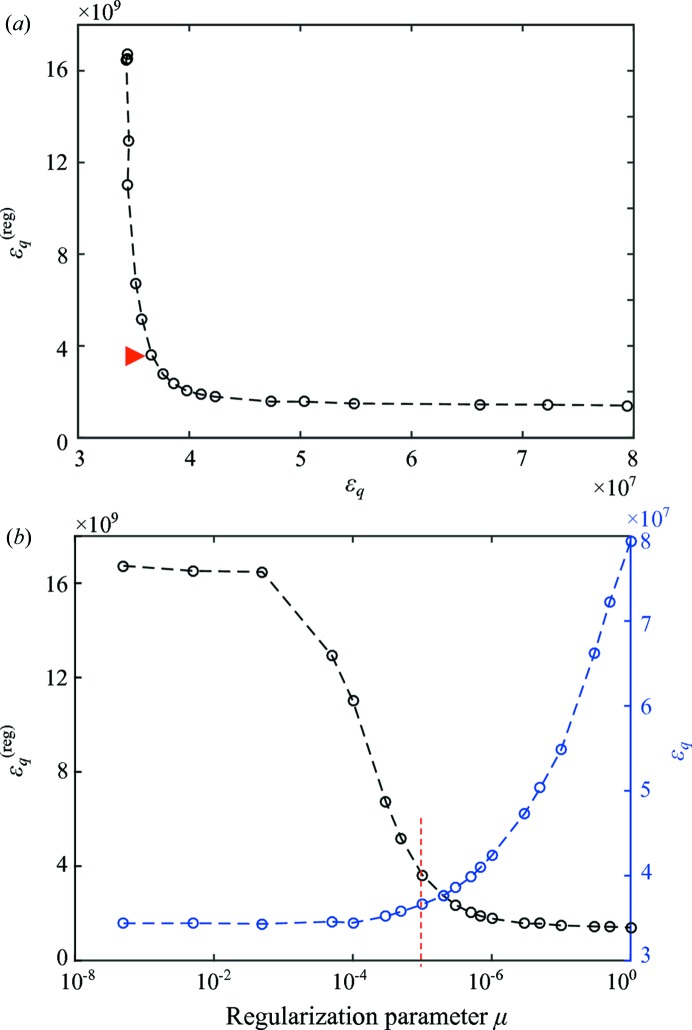
(*a*) L-curve used to find an appropriate regularization parameter μ. (*b*) shows the dependence of the penalty term 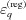 (left black axis) and of the of error metric 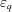 (right blue axis) on the regularization parameter μ. These two values are combined in the L-curve. The red arrow and red dashed line indicate the regularization parameter selected for this trabecular bone sample as explained in the text.

In order to select an appropriate regularization parameter μ the L-curve technique was applied (Hansen, 1992 ▸; Li *et al.*, 2003 ▸; Belge *et al.*, 2002 ▸; Santos & Bassrei, 2007 ▸). The L-curve is a plot of the data error, 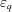, *versus* the regularization penalty term, 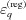, where each point corresponds to different values of the regularization strength, μ (see Fig. 11 ▸ *a*). The corner of the L-curve corresponds to a trade-off between a smooth solution with a high error metric 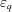 and a solution with a small error but more high-frequency noise (Hansen, 1992 ▸). Fig. 11 ▸(*b*) shows the two terms of the error metric 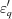 *versus* the regularization parameter μ. The regularization parameter should be selected around the inflection point combined with a visual quality inspection of the solution (Fig. 10 ▸). Here we chose 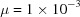. This point is on the left side corner of the L-curve (red arrow in Fig. 11 ▸ *a*) because we prioritize a small error over a smooth solution.

**Figure 12:**
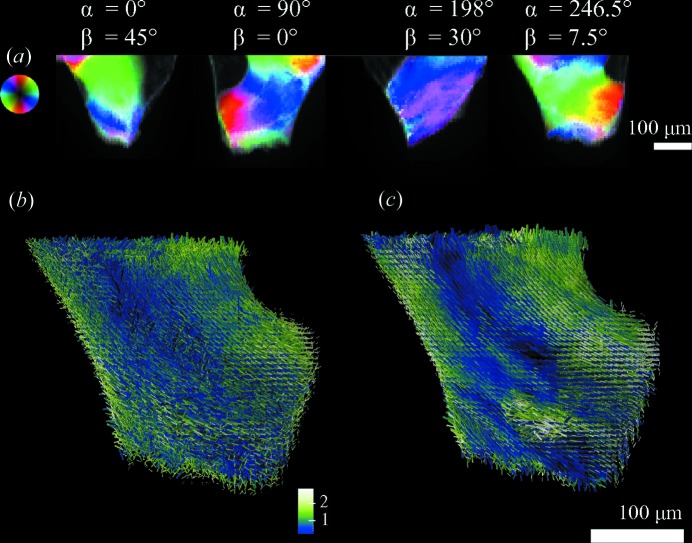
Orientation of the bone ultrastructure from a human trabecular bone (sample C). Four two-dimensional SAXS projections at different sample orientations are shown in (*a*), where the colour wheel represents the main scattering orientation, the hue the scattering intensity and the degree of orientation the colour saturation. The orientation of the bone ultrastructure retrieved from SAXS tensor tomography is shown in (*b*) and (*c*), where the colour represents the degree of orientation and the length of the isotropic component 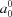. (*b*) Reconstruction without regularization applied and (*c*) with regularization of the spherical harmonics coefficients 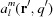 and on the direction 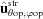 as described in the text.

A three-dimensional visualization of the resulting SAXS tensor tomography reconstruction, corresponding to Fig. 10 ▸(*a*), is shown in Fig. 12 ▸(*b*). The reconstruction using both regularization of the coefficients, with a 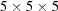 Hamming window, and of the orientation with 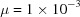 is shown in Fig. 12 ▸(*c*). The reconstruction without regularization shows substantial noise particularly in the orientations, and no clear domains are visible. This variation is not supported by the measured data, as shown in projections from different sample orientations (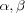) in Fig. 12 ▸(*a*). The regularization with 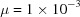 as shown in Fig. 12 ▸(*c*) reduced the high-frequency noise without suppressing the different orientations found in the sample and leads to better defined regions of a higher degree of orientation (light green). The sample contains different domains of orientation, each spanning some tens of micrometres, similar to the sample shown in Figs. 7 ▸ and 9 ▸. The higher resolution obtained here with a smaller beam size of 5 × 1.4 µm enables us to resolve the transition region between the domains.

## 6. Conclusion   

SAXS tensor tomography aims at reconstructing the local three-dimensional reciprocal-space map for each volume element within a three-dimensional sample. This can be achieved through gradient-based optimization. An adequate numerical representation of the three-dimensional reciprocal-space map, for which only a few quantities or coefficients have to be recovered for each voxel, can be critical towards developing an approach that is efficient both in computational and measurement time. Liebi *et al.* (2015 ▸) introduced this reconstruction approach using spherical harmonics as a base to represent the reciprocal-space map and demonstrated it with a millimetre-sized sample of trabecular bone. The three-dimensional reciprocal-space map comprises information on the main orientation of the nanostructure for different *q* ranges and also its degree of orientation. The reciprocal-space map could further be used as input for fitting the underlying nanostructure, similarly to what has been done on two-dimensional SAXS data, for example to retrieve size parameters of the mineralized platelets in bone (Fratzl *et al.*, 2005 ▸; Turunen *et al.*, 2016 ▸).

In this article we present a detailed description of the algorithm as well as a validation study to confirm the suitability of spherical harmonic coefficients to represent the three-dimensional reciprocal-space map of trabecular bone where the features of the mineralized collagen fibrils can be recovered. In order to reduce the number of coefficients that need to be optimized we provide for each voxel a parameterization of the spherical harmonic zenith direction, which provides directly the orientation of the main symmetry axis of the nanostructure in three dimensions and subsequently allows us to impose reciprocal-space symmetry constraints. Regularization strategies for both the coefficients and the orientation are introduced and described in detail. An important consideration is the distribution of projection angles for the measurements. By selectively removing angles the effect on the reconstruction is shown, which confirms that a uniform distribution of sample orientations is of significant benefit for the reconstruction quality.

SAXS tensor tomography provides a technique that can probe three-dimensional nanostructure information in relatively large volumes, offering the unique chance to correlate spatial nanoscale features over several millimetres, *i.e.* separated over five or six orders of magnitude. The technique is applicable to a wide range of specimens in biology and materials science and also scalable. While the original demonstration was with 20 µm voxel size, by using microfocusing optics and improved scanning hardware this article demonstrated the technique to a spatial resolution of 5 µm. By suitable changes in optics and hardware the resolution can be adjusted to the application of interest.

## Appendix A Analytical expressions for the gradients

The gradient of the error metric , as defined in equation (4), with respect to the spherical harmonic coefficients is given by

where the notation  indicates that the function is evaluated at the detector plane, , in the laboratory coordinates , and with . Notice that the first term in brackets in equation (10) is only a function of  in the laboratory coordinates  and that it is constant along the *z* direction, which is effectively a back-projection. This is then computed *via* back-projection along the beam-propagation direction using bilinear interpolation.

The gradient with respect to  is given by

The derivatives of the spherical harmonic functions are given by (Wolfram Research Inc., 2017*a* ▸,*b* ▸)

Using equation (2)

Similarly, the gradient with respect to  is given by

with

Using this gradient and the previous search direction a conjugate gradient direction is defined, and along this direction a line search is carried out to look for the minimum value of the error metric (Press *et al.*, 2007 ▸).

If the smooth penalty term  is introduced in the error metric as described in equations (5) and (6), the gradient of the error metric with respect to the spherical harmonic co­efficients [equation (10)] is

with

If regularization of the local preferential orientation  is used, the gradient with respect to  is

with

with

Similarly, expressions for the terms with finite differences in the *y* and *z* directions can be obtained to complete equation (19). Equivalent expressions can be found for the gradient with respect to .

## Appendix B Experimental details

### b1. Sample preparation

Trabeculas from a 12th thoracic (T12) human vertebra from a 50-year-old woman (sample A) and a 73-year-old man (samples B and C) were extracted and soft tissue was removed before embedding into polymethyl methacrylate (PMMA). The vertebrae were obtained from the Department of Anatomy, Histology and Embryology at the Innsbruck Medical University, Innsbruck, Austria, with the written consent of the donors according to Austrian law. All of the following procedures were performed in accordance with Swiss law, the Guideline on Bio-Banking of the Swiss Academy of Medical Science (2006) and the Swiss ordinance 814.912 (2012) on the contained use of organisms. To obtain sample A, a 20 µm-thick section was cut using a microtome (HM 355S; Thermo Fisher Scientific Inc., USA) and mounted on 12 µm-thick Kapton adhesive tape (T2000023; Benetec, Wettswil, Switzerland). A cylinder of about 1 mm and 250 µm diameter was milled out of the PMMA block for samples B and C, respectively.

### b2. Experiments

Measurements were performed at the coherent small-angle X-ray scattering (cSAXS) beamline (X12SA) of the Swiss Light Source, Paul Scherrer Institut, Switzerland. The X-ray beam was monochromated by a fixed-exit double-crystal Si(111) monochromator to 12.4 keV for samples A and B, and to 11.2 keV X-ray energy for sample C. Two different types of focusing were used. For samples A and B the beam was focused horizontally by bending the second monochromator crystal and vertically by bending a Rh-coated mirror resulting in a beam size of about 20 × 20 µm and 25 × 25 µm, respectively. For sample C the beam was focused with a Fresnel zone plate to a beam size of 5 × 1.4 µm (Lebugle *et al.*, 2017 ▸). To minimize air scattering and X-ray absorption a 7 m (samples A and B) or a 2 m long (sample C) flight tube was placed between the sample and detector. X-ray scattering was measured with a Pilatus 2M detector (Kraft *et al.*, 2009 ▸) and the transmitted-beam intensity with a photo-diode mounted on a beamstop placed inside the flight tube.

Sample A, a thin section of trabecular bone, was measured in the three-dimensional scanning SAXS setup as described by Georgiadis *et al.* (2015 ▸) and schematically shown in Fig. 1 ▸(*a*). The sample was mounted on a goniometer placed on a rotation stage on top of a two-axis translation stage. The sample was continuously moved in the *x* direction while SAXS patterns were measured with 50 ms exposure time. The scan was repeated at 30 different sample orientations around the *y* axis in steps of 10°, omitting the angles where the section was close to parallel to the X-ray beam, *i.e.* 80–100° and 260–280°. The scan step in *x* was chosen to be  µm to have a consistent number of scanning points across the specimen. The scan step in *y* was 20 µm. To identify the scattering that originates from a specific voxel under all sample rotation angles, image registration (Guizar-Sicairos *et al.*, 2008 ▸) on the transmission images was used, as described in detail by Georgiadis *et al.* (2015 ▸).

Samples B and C were measured with the three-dimensional tensor tomography setup as described by Liebi *et al.* (2015 ▸) and schematically shown in Fig. 1 ▸(*b*). The sample was mounted on a goniometer on two perpendicular rotation stages which were placed on a two-axis scanning stage.

Sample B was measured at  projections, each with  scanning steps with a step size of 25 µm. The sample was continuously moved in the *x* direction while SAXS patterns were measured with an exposure time of 30 ms, which was limited by the detector read-out. The angular spacing was 15° between −30° and 45° in β and 4.5° between 0° and 180° in α. The total exposure time was 8.3 h. Each voxel was exposed for a total of 7.2 s. Because of overhead from motor movement, the total measurement time was 20.3 h.

Sample C was measured at  projections, each with  scanning steps with a step size of 5 µm and an exposure time of 50 ms. As the beam in the vertical (*y*) was smaller than the step size, the sample was continuously moved in the *y* direction during measurement in order to probe the whole sample volume. Based on our findings with regard to the advantage of an even distribution of projection angles  = 7.5° and  = 7.5°  have been used. The total exposure time was 11.8 h and the total measurement time 21.3 h. The overhead of motor movements was reduced compared with the measurement of sample B, mainly by introducing a snake-like scan pattern where subsequent lines are scanned in opposite directions, which reduces the time the motor needs to reach the line scan starting position. Additional projections were measured at , achieving  = 3° to have enough projections for the standard filtered back-projection reconstruction used for image registration as described in §5.

### b3. Availability of the software

A current version of the data analysis software is available upon request from the corresponding authors.
